# Association of perceived job insecurity with ischemic heart disease and antihypertensive medication in the Danish Work Environment Cohort Study 1990–2010

**DOI:** 10.1007/s00420-015-1030-5

**Published:** 2015-03-03

**Authors:** Ute Latza, Karin Rossnagel, Harald Hannerz, Hermann Burr, Sylvia Jankowiak, Eva-Maria Backé

**Affiliations:** 1 Division Work and Health, Federal Institute for Occupational Safety and Health (BAuA), Noeldnerstr. 40/42, 10317 Berlin, Germany; 2 National Research Centre for the Working Environment (NRCWE), Lersø Parkallé 105, 2100 Copenhagen Ø, Denmark

**Keywords:** Psychosocial work factor, Job insecurity, Cardiovascular risk factor, Antihypertensive medication, Ischemic heart disease

## Abstract

**Purpose:**

To determine the effect of job insecurity based on repeated measurements on ischemic heart disease (IHD) and on antihypertensive medication.

**Methods:**

The study population consists of 12,559 employees aged 18–59 years of the Danish Work Environment Cohort Study. With an open cohort design, data from up to four representative waves were linked to four registers. Poisson regression with time-dependent covariates was used to estimate the rate ratio (RR) with confidence interval (CI) of perceived job insecurity associated with first-time IHD hospitalization or mortality 1991–2010 (*n* = 561 cases) and incident dispensing of prescribed antihypertensive medications 1996–2010 (*n* = 2,402 cases).

**Results:**

Participants with perceived job insecurity filled more antihypertensive prescriptions (age-, gender-, and calendar year-adjusted RR 1.23, 95 % CI 1.12–1.33) and had a borderline significant higher IHD incidence (RR 1.23, 95 % CI 0.98–1.55). In a subanalysis, the risk of antihypertensive medication dispensed was only significant among employees with worries about both unemployment and poor reemployment opportunities. After explorative stratifications by age, gender, and occupational status, perceived job insecurity was associated with more dispensing of antihypertensive medications to participants less than 50 years of age.

**Conclusions:**

In a country with high social security and active labor market policy, employees with the feeling of an insecure job have a modestly increased risk to fill an antihypertensive prescription. Further studies on health risks of job insecurity should consider improved exposure assessment, earlier outcomes such as medication in order to increase statistical power, and identification of vulnerable population groups.

## Background

The relevance of occupational exposures (e.g., job stress, fine particulate dust, noise, shift work, and environmental tobacco smoke) for the prevention of both cardiovascular diseases (CVDs) in the general population and at the workplace is often underestimated (Cullen 2009). Among potential risk factors at the workplace, psychosocial factors play an increasingly important role. The most recent meta-analysis (Kivimäki et al. 2012) and systematic review (Backé et al. 2012) further support the association between work stressors [mainly based on the models of Karasek et al. (1998) and Siegrist et al. (1990)] and CVD, in particular ischemic heart disease (IHD).

Job insecurity has gained importance within the frame of a rapidly changing organizational work environment characterized by restructuring and downsizing combined with unpredictable economic situations (Vahtera and Virtanen 2013).

The construct of job insecurity itself has undergone a change of meaning. During the 1960s and 1970s, job security was often to be found in larger inventories of work climate in the USA and was regarded as a motivational factor (reviewed by Sverke et al. 2002). In the mid-1980s, research began to focus on job insecurity and along with this came a change in the meaning from being seen as a motivator to being defined as a stressor (Greenhalgh and Rosenblatt 1984).

The association of job insecurity and CVD has been investigated in cohort studies (Siegrist et al. 1990; Lee et al. 2004; Slopen et al. 2012; Ferrie et al. 2013; Netterstrøm et al. 2010) and a meta-analysis of these and hitherto unpublished European cohorts (Virtanen et al. 2013). To our knowledge, all available cohort studies consider single baseline measurements of job insecurity and late outcomes such as hospitalization or mortality.

In a recent systematic review, Pejtersen et al. (2014) demonstrated low statistical power of most analyses on work-related psychosocial factors and incidence of IHD. Thus, there is a need to evaluate more frequently occurring outcomes that are highly associated with IHD. Further, the comparatively late outcome of IHD mortality and/or morbidity favors bias due to the healthy worker effect. The most important risk factor in the multifactorial etiology of CVD is hypertension (Perk et al. 2012) which can also be regarded as an own disease entity. Evidence from a systematic review for a positive association with hypertension is available for job strain (Babu et al. 2014). So far, only one longitudinal study on job insecurity with self-reported outcome and ambiguous results is available. Job insecurity was a predictor of incident use of antihypertensive medications only among males in the population-based sample of 2,357 adults free of self-reported hypertension or high blood pressure (Levenstein et al. 2001).

The Danish Work Environment Cohort Study (DWECS) offers the possibility to investigate perceived job insecurity in a large representative sample of the working population with a long follow-up. The questionnaire data can be linked to diverse population-based registers.

Previous analyses of perceived job insecurity in the DWECS investigated self-rated health (Rugulies et al. 2008), and indicators of depression, e.g., use of antidepressants (Rugulies et al. 2010). Further, job insecurity was considered as a covariate in an analysis of shift work and circulatory diseases (Tüchsen et al. 2006).

The aim of the study was to determine the effect of job insecurity (defined by perceived threat of unemployment and/or perceived lack of reemployment opportunities based on time-dependent exposure measurements) on the incidence of IHD (measured by first-time hospitalization or mortality due to IHD) and the incident dispensing of prescribed antihypertensive medications. Subanalyses for the identification of vulnerable subpopulations with stratifications by gender, age, and occupational status and a subanalysis with an alternate operationalization of job insecurity were performed.

## Materials and methods

### Study population

In DWECS, a representative sample of inhabitants of Denmark, aged 18–59 years, was interviewed in 1990, with a response rate of 90 %. The 1990 panel consisted of a simple random sample drawn on October 1, 1990, from the central population register (1/330 of the national population). In 1995, 2000, and 2005, everyone was contacted again (response rates of 80, 75, and 63 %, respectively), disregarding previous participation or employee status. The design is described in detail elsewhere (Burr et al. 2003; Feveile et al. 2007).

The study population consists of all employees who participated in the DWECS waves in the years 1990, 1995, 2000, and 2005 with an open cohort design and were at least 21 years old at the start of their first follow-up. Overall 12,559 subjects were included (6,061 men, and 6,498 women). Nearly half of the participants (*n* = 5,742) entered the cohort in 1990 (with a start of follow-up in 1991) (Table 1). For another 36 %, follow-up started in 2006. Most person-years accumulated between 2006 and 2010 (37.3 %). About a third of subjects (39.1 %) participated in only one survey and another third in all four DWECS waves (29.0 %). The mean length of follow-up for IHD was 8.9 years for men, 8.4 years for women, and 8.7 years for both genders combined (range 0–20 years). The mean length of follow-up for antihypertensive medicine was 7.5 years for men, 7.1 years for women, and 7.3 years for both genders combined (range 0–15 years).

**Table 1:** Selected characteristics of participants in the DWECS between 1990 and 2010 by gender

Variable and level	Men	Women	Both
	*Number (percent)*		
Calendar year of start of follow-up			
1991	2,907 (48.0 %)	2,835 (43.6 %)	5,742 (45.7 %)
1996	601 (9.9 %)	659 (10.1 %)	1,260 (10.0 %)
2001	477 (7.9 %)	562 (8.7 %)	1,039 (8.3 %)
2006	2,076 (34.3 %)	2,442 (37.6 %)	4,518 (36.0 %)
Number of repeated measures (DWECS waves)			
1	2,359 (38.9 %)	2,557 (39.4 %)	4,916 (39.1 %)
2	849 (14.0 %)	783 (12.1 %)	1,632 (13.0 %)
3	1,148 (18.9 %)	1,217 (18.7 %)	2,365 (18.8 %)
4	1,705 (28.1 %)	1,941 (29.9 %)	3,646 (29.0 %)
First-time hospitalization			
Cardiovascular disease^a^	417 (6.9 %)	228 (3.5 %)	645 (5.1 %)
Ischemic heart disease^b^	333 (5.5 %)	163 (2.5 %)	496 (3.9 %)
Death			
Cardiovascular disease^a^	59 (1.0 %)	18 (0.3 %)	77 (0.6 %)
Ischemic heart disease^b^	54 (0.9 %)	11 (0.2 %)	65 (0.5 %)
Emigration	209 (3.5 %)	156 (2.4 %)	365 (2.9 %)
	*Person-years at risk (percent)*		
Calendar period			
1991–1995	10,785 (19.9 %)	10,071 (18.4 %)	20,856 (19.2 %)
1996–2000	12,355 (22.8 %)	11,650 (21.3 %)	24,005 (22.1 %)
2001–2005	11,653 (21.5 %)	11,675 (21.3 %)	23,328 (21.4 %)
2006–2010	19,340 (35.7 %)	21,298 (38.9 %)	40,639 (37.3 %)
Age (years)			
<40	19,863 (36.7 %)	18,356 (33.6 %)	38,219 (35.1 %)
40–49	15,982 (29.5 %)	17,526 (32.0 %)	33,508 (30.8 %)
50–59	13,375 (24.7 %)	14,455 (26.4 %)	27,830 (25.6 %)
60–69	4,793 (8.9 %)	4,288 (7.8 %)	9,081 (8.3 %)
70+	122 (0.2 %)	68 (0.1 %)	190 (0.2 %)
BMI (kg/m^2^)			
<25	28,493 (52.6 %)	40,247 (73.6 %)	68,740 (63.2 %)
25 to < 30	20,919 (38.6 %)	11,100 (20.3 %)	32,018 (29.4 %)
≥30	4,722 (8.7 %)	3,347 (6.1 %)	8,069 (7.4 %)
Smoking status			
Never smoker	19,299 (35.7 %)	22,681 (41.5 %)	41,980 (38.6 %)
Ex-smoker	13,057 (24.1 %)	12,510 (22.9 %)	25,566 (23.5 %)
Current smoker	21,778 (40.2 %)	19,503 (35.7 %)	41,281 (37.9 %)
SES^c^			
Executive managers and academics	11,163 (20.6 %)	5,554 (10.2 %)	16,717 (15.4 %)
Middle managers and persons with 3–4 years of higher education	8,007 (14.8 %)	13,257 (24.2 %)	21,265 (19.5 %)
Other white-collar workers	13,035 (24.1 %)	22,934 (41.9 %)	35,969 (33.1 %)
Skilled blue-collar workers	10,114 (18.7 %)	3,994 (7.3 %)	14,107 (13.0 %)
Semiskilled or unskilled workers	11,815 (21.8 %)	8,955 (16.4 %)	20,770 (19.1 %)
Shift work			
Fixed day shift	44,430 (82.1 %)	44,190 (80.8 %)	88,620 (81.4 %)
All others	9,704 (17.9 %)	10,503 (19.2 %)	20,208 (18.6 %)
Perceived job insecurity			
Yes	18,126 (33.5 %)	20,837 (38.1 %)	38,963 (35.8 %)
No	36,008 (66.5 %)	33,856 (61.9 %)	69,864 (64.2 %)

Danish Work Environment Cohort Study

^a^Cardiovascular disease (CVD, extended outcome): ICD-10 = I20–I25, I63, I65, I66, I70, and ICD-8 = 410–414, 432–434, 440

^b^Ischemic heart disease (IHD): ICD-10 = I20–25, and ICD-8 = 410–414

^c^Socioeconomic status

The included persons were first followed from January 1, 1991, until any of the following events occur: (S)he had a hospital contact with the sought outcome as principal diagnosis, (s)he emigrated, (s)he dies, or the study period ended (31 December 2010). In a second sequence, the study population was followed from January 1, 1996, for incident dispensing of prescribed antihypertensive medications, emigration, death, or the end of the study period. Only those who were free from the clinical endpoint of the respective follow-ups, throughout the calendar year preceding baseline, were included in the analyses.

### Ethics approval

The study complies with The Act on Processing of Personal Data (Act No. 429 of 31 May 2000), which implements the European Union Directive 95/46/EC on the protection of individuals. The data usage was approved by the Danish National Institute for Health Data and Disease Control, Statistics Denmark (project number 705743), and the Danish Data Protection Agency (file number 2012-54-0042). The required approvals were obtained for two authors (H.H. and K.R.).

### Cardiovascular disease endpoints

For outcome ascertainment, DWECS data were linked via a personal identification number (PIN) to the following four population-based Danish registers: (a) the Civil Registration System (CRS) (Pedersen 2011), (b) the Cause of Death Register (Helweg-Larsen 2011), (c) the National Patient Register (Lynge et al. 2011), and (d) the Danish National Prescription Register (DNPR) (Kildemoes et al. 2011).

The CRS contains the PIN and information on gender, addresses, and dates of birth, death, and migrations for every person who is, or has been, an inhabitant of Denmark sometime between 1968 and the present. The National Patient Register contains data from all public hospitals in Denmark (more than 99 % of all admissions). Until 1994, the register only included inpatients, but from 1995 it also covers outpatients and emergency ward visits.

The diagnoses were coded according to ICD-8 and since 1994 to ICD-10. In the DNPR, all prescribed medications (as opposed to over-the-counter products) dispensed at pharmacies in Denmark are reported since 1995 (Kildemoes et al. 2011).

Prescriptions are coded in accordance with the Anatomical Therapeutic Chemical (ATC) system.

*Combined first-time IHD hospitalization or IHD mortality*: The case definition included the ICD-10 codes I20–I25 and the corresponding ICD-8 codes (410–414).

*CVD*: The extended outcome in the subanalysis comprised of IHD, and cerebrovascular diseases (I63, I65, I66), and I70 (or the corresponding ICD-8 codes 410–414, 432–434, 440). Transient ischemic attack (ICD-10 G45) was not included due to potential serious misclassification (Johnsen et al. 2002).

*Incident dispensing of prescribed antihypertensive medication*: The case definition includes the incident dispensing of the following prescribed ATC codes: C02 antihypertensiva, C03 diuretics, C07 alpha- and beta-blockers, C08 calcium channel blockers, C09 ACE inhibitors, and angiotensin-II antagonists. From DNPR, the variables PIN, ATC code, and date of sale are used. In a validation study (Hannerz et al. 2014), these ATC codes showed similar correlations with socioeconomic groups as hospital treatment or death due to IHD.

### Questionnaire data

From DWECS, the variables personal identification number, and self-reported information on the independent variable and covariates are utilized (for categorizations, see Table 1).

### Independent variable

Perceived job insecurity is based on two out of the four items of the job insecurity scale from the Copenhagen Psychosocial Questionnaire (COPSOQ I) with binary answering options. Participants are categorized as having job insecurity if they have answered “yes” to at least one of the two items covering aspects of job loss: “Are you worried about becoming unemployed?” and “Do you worry that it will be difficult for you to find a new job with your present qualifications?” (Pejtersen et al. 2010).

The correlation between the two job insecurity items was low (Spearman’s correlation coefficient 0.34838, 0.26765, 0.31001, and 0.38048 in 1990, 1995, 2000, and 2005, respectively) but statistically significant (all *p* < 0.0001).

### Covariates

Body mass index (BMI) is calculated from self-reported weight and height as kg/m^2^. Socioeconomic status (SES) is defined based on employment grade, job title, and education (Borg and Kristensen 2000). Smoking status is obtained from the question “Do you smoke?” (Yes/I have smoked, but not any more/I have never smoked). The variable shift work (fixed day shift vs. all others) was assigned the value “fixed day shift” if the person answered with the first response category to the question: “What kind of work schedule do you have? (permanent day duty/two shifts/three shifts/fluctuating according to special schedule or rotation/permanent evening duty/permanent night duty/permanent morning duty/other)”as described (Tüchsen et al. 2006).

### Statistical models

Repeated measurements of job insecurity and the covariates from up to four DWECS waves were utilized to account for time-dependent changes of information (Breslow and Day 1987). Poisson regression with ungrouped data and job insecurity as time-dependent covariate was used (PROC GENMOD procedure of SAS 9.3; SAS Institute Inc., Cary, NC, USA) to estimate rate ratios (RRs) with 95 % confidence intervals (CIs) for each of the two endpoints (a) incidence of combined first-time IHD hospitalization or IHD mortality with a subanalysis for CVD as extended outcome definition and (b) incident antihypertensive medications dispensed in order to increase statistical power. Briefly, follow-up time is stratified by calendar year and repeated measurements used strata-wise with last available information carried forward (e.g., information from DWECS wave 1990 is used for 1991–1995 and then information from 1995 for 1996–2000, etc.).

The minimally adjusted model includes age (<40, 40–49, 50–59, 60–69, 70+ years), gender (male, female), and calendar year of survey (1990, 1995, 2000, 2005). Multivariate models additionally considered BMI (<25, 25 to <30, ≥30 kg/m^2^) as biological covariate and smoking status (never, former, current smoker) as behavioral covariate. Alcohol consumption and physical activity in leisure time were only included in the DWECS questionnaires of 2000 and 2005 and therefore omitted. Questions on pack years, and clinical measurements of blood lipids or glucose levels and blood pressure were not available. Work-related covariates included SES (executive managers and academics, middle managers and persons with 3–4 years of higher education, other white-collar workers, skilled blue-collar workers, semiskilled or unskilled (blue collar) workers) and shift work (fixed day duty vs. all others).

Analyses by gender were performed (nested hypothesis testing), if the minimally adjusted risk estimates for the association between perceived job insecurity and CVD and antihypertensive medications, respectively, were significant.

Based on a priori statistical power calculations, further subanalyses were possible for antihypertensive medications dispensed. For hypothesis-generating purposes, the minimally adjusted models were additionally stratified for categorized age at baseline (<50, ≥50 years) and occupational status (white collar = executive managers and academics, middle managers and persons with 3–4 years of higher education, other white-collar workers versus blue collar = skilled blue-collar workers, semiskilled or unskilled workers). In order to examine a potential dose effect, the independent variable was recoded into a new variable of perceived risk of unemployment and anticipated reemployment opportunities with three categories (a) not worried about becoming unemployed, (b) worried about becoming unemployed and not worried having difficulty in finding another job with the present qualifications, and (c) worried about becoming unemployed and worried about having difficulty in getting another job with the present qualifications (Rugulies et al. 2008).

Missing values on considered covariates varied between *N* = 10 (smoking status in the 1995 wave) and *N* = 264 (shift work in 1990 wave) and were generally higher for shift work, for SES, and for the 1990 wave. If information on SES or shift work was missing in one wave, then the missing value was replaced with the latest available SES/shift work category. Missing values for BMI and smoking were replaced assuming the least favorable exposure if necessary. If BMI was missing in one wave but non-missing in the neighboring wave(s), then the missing value was replaced by the average of the non-missing neighboring values.

## Results

The main outcome of first-time hospitalization due to IHD (3.9 %) or IHD mortality (0.5 %) had 561 incident cases and the extended outcome CVD 722 incident cases. A third of person-years were contributed to overweight (29.4 %), in particular men (38.6 %), and 7.4 % with obesity (BMI ≥ 30). More than a third of participants (38.6 %), particularly females, never smoked while being at risk. Vertical occupational segregation was present with concentration of males in higher categories of the professional hierarchy (20.6 % of person-years as executive managers and academics among male and 10.2 % among female participants, respectively). Women mainly contributed to person-years as white-collar workers (41.9 %) and men frequently as skilled blue-collar workers (18.7 %) indicating horizontal segregation.

Since data on the dispensing of prescribed antihypertensive medications have been available only from 1995 onwards, follow-up for participants with no antihypertensive medications dispensed in 1995 started in 1996 with a maximum of three DWECS waves (55 % had ≥2 waves). Twenty-one percent of participants (2,402 out of 11,671) were registered with incident antihypertensive medications dispensed between 1996 and 2010.

Job insecurity was reported on average by 35.8 % of all participants. Overall perceived job insecurity decreased from 44.7 % in 1990 to 31.2 % in 2000 and was stable from then on (31.9 % in 2005). An analysis of temporal trends in perceived job insecurity by gender is shown in Table 2 (minimum: men 1995 for (a) 16.4 %; maximum: women 1990 for (a) and/or (b) 49.8 %). In the 1990 wave, more women were worried about becoming unemployed than men (33.9 vs. 27.9 %) and were worried about poor reemployment opportunities (37.1 vs. 25.5 %). Gender differences in the frequency of perceived job insecurity leveled out in the following DWECS waves; men and women were similar in 2005.

**Table 2:** Temporal variation in the frequency of job insecurity items by gender in Denmark 1990–2005

Perceived job insecurity (items)	Number (%) for men, women, and both genders			
	1990	1995	2000	2005
(a) Perceived threat of unemployment (worried about becoming unemployed)	Men: 809 (27.9 %)	Men: 432 (16.4 %)	Men: 452 (18.1 %)	Men: 682 (18.1 %)
	Women: 901 (33.3 %)	Women: 453 (18.7 %)	Women: 435 (17.2 %)	Women: 787 (18.9 %)
	Both: 1,710 (30.5 %)	Both: 885 (17.5 %)	Both: 887 (17.7 %)	Both: 1,469 (18.5 %)
(b) Perceived lack of reemployment opportunities (worried about having difficulty in finding a new job with the present qualifications)	Men: 739 (25.5 %)	Men: 594 (22.7 %)	Men: 488 (19.6 %)	Men: 871 (23.3 %)
	Women: 1,001 (37.1 %)	Women: 715 (29.5 %)	Women: 636 (25.2 %)	Women: 1,056 (25.5 %)
	Both: 1,740 (31.1 %)	Both: 1,309 (26.0 %)	Both: 1,124 (22.4 %)	Both: 1,927 (24.4 %)
Variable job insecurity (a) and/or (b)	Men: 1,154 (39.9 %)	Men: 822 (31.4 %)	Men: 740 (29.7 %)	Men: 1,157 (31.0 %)
	Women: 1,342 (49.8 %)	Women: 913 (37.8 %)	Women: 822 (32.6 %)	Women: 1,346 (32.6 %)
	Both: 2496 (44.7 %)	Both: 1,735 (34.5 %)	Both: 1,562 (31.2 %)	Both: 2,503 (31.9 %)

### Perceived job insecurity and first-time hospitalization or mortality

The combined risk of first-time hospitalization or mortality due to IHD was slightly increased, but the confidence intervals were wide (RR 1.23, 95 % CI 0.98–1.55 after adjustment for age, gender, and calendar year) (Table 3). After further adjustment for all covariates, the risk was attenuated and the confidence interval wider (RR 1.19, 95 % CI 0.94–1.51 with additional adjustment for BMI, smoking, SES, and shift work).

**Table 3:** Rate ratio (employees with perceived job insecurity vs. employees without job insecurity) for combined first-time hospitalization or mortality due to IHD in Denmark 1991–2010

Covariables	Rate ratio (95 % confidence interval) of IHD
Age, gender, calendar year^a^	1.23 (0.98–1.55)
+BMI^b^	1.22 (0.97–1.54)
+Smoking	1.22 (0.96–1.53)
+SES^c^, shift work	1.20 (0.95–1.52)
Age, gender, calendar year, BMI, smoking, SES, shift work	1.19 (0.94–1.51)

Ischemic heart disease ICD-10 = I20–25, and ICD-8 = 410–414

^a^Minimally adjusted model

^b^Body mass index

^c^Socioeconomic status

In the subanalysis for the extended outcome CVD, the risk associated with perceived job insecurity was even lower than for IHD and the confidence intervals remained wide (not shown).

### Perceived job insecurity and incident dispensing of prescribed antihypertensive medications

The risk of incident dispensing of prescribed antihypertensive medications was increased (RR 1.23, 95 % CI 1.12–1.35 after adjustment for age, and calendar year) with minor gender differences (men: adjusted RR 1.26, 95 % CI 1.10–1.46 and women: adjusted RR 1.19, 95 % CI 1.04–1.36) (Table 4). After adjustment for all covariates, the risk was attenuated and the confidence intervals wider (RR 1.18, 95 % CI 1.07–1.31).

**Table 4:** Rate ratio (employees with perceived job insecurity vs. employees without job insecurity) for incident dispensing of prescribed antihypertensive medications^a^ in Denmark 1996–2010

Covariables	Rate ratio (95 % confidence interval) of antihypertensive medications^a^		
	Men	Women	Both
Age, (gender), calendar year^b^	1.26 (1.10–1.46)	1.19 (1.04–1.36)	1.23 (1.12–1.35)
+BMI^c^	1.26 (1.10–1.45)	1.15 (1.01–1.32)	1.21 (1.10–1.33)
+Smoking	1.26 (1.08–1.43)	1.18 (1.03–1.34)	1.22 (1.11–1.34)
+SES^d^, shift work	1.24 (1.08–1.43)	1.15 (1.01–1.32)	1.20 (1.09–1.32)
Age, (gender), calendar year, BMI, smoking, SES, shift work	1.25 (1.08–1.44)	1.12 (0.98–1.29)	1.18 (1.07–1.31)

^a^ATC codes: C02 antihypertensiva, C03 diuretics, C07 alpha- and beta-blockers, C08 calcium channel blockers, C09 ACE inhibitors, and angiotensin-II antagonists

^b^Minimally adjusted model

^c^Body mass index

^d^Socioeconomic status

In a subanalysis with investigation of a potential dose effect of the two items of perceived job insecurity, the risk of incident antihypertensive medications dispensed was only significant for subjects who were worried about becoming unemployed and worried about reemployment opportunities (adjusted RR 1.27, 95 % CI 1.10–1.47) compared with subjects who were not worried about becoming unemployed. The risk for subjects who were worried about becoming unemployed, but not worried about poor reemployment opportunities was lower and not significant (adjusted RR 1.19, 95 % CI 0.99–1.42).

In subanalyses with stratification for occupational status, and age at baseline (not shown), the risk of antihypertensive medication was higher for participants of less than 50 years than for older subjects (<50 years adjusted RR 1.41, 95 % CI 1.22–1.63; ≥50 years: adjusted RR 1.10, 95 % CI 0.97–1.25). Blue-collar workers with perceived job insecurity filled more antihypertensive prescriptions at pharmacies (adjusted RR 1.29, 95 % CI 1.09–1.52) than white-collar workers (adjusted RR 1.18, 95 % CI 1.05–1.33) with a large overlap in the confidence intervals.

## Discussion

Perceived job insecurity was associated with incident dispensing of prescribed antihypertensive medications. The risk of hospitalization or death due to CVD associated with job insecurity was similar but only borderline significant. In an exploratory subanalysis, merely subjects with perceived threat of job loss together with anticipated lack of reemployment opportunities filled antihypertensive prescriptions and not subjects with perceived lack of reemployment opportunities only. Further subanalyses with stratifications by age, gender, and occupational status provided some indication of a higher risk for younger participants.

### Comparison with other studies

As compared to the previous cohorts with only a single baseline measurement summarized by Virtanen et al. (2013), the use of time-varying measurements of job insecurity (and other covariates) from up to four surveys minimized exposure misclassification. The consideration of incident dispensing of prescribed antihypertensive medications as a second outcome (*n* = 2,402 cases) increased the statistical power and allowed for subanalyses. The DWECS contributed to the meta-analysis with the 1990 wave and 45 incident IHD events (Virtanen et al. 2013), as compared to the open cohort design of the present DWECS analysis with four waves and 561 incident IHD events.

The modestly increased risk estimates of 1.23 in the present study for the associations of perceived job insecurity with both dispensing of antihypertensive medications and IHD hospitalization or mortality correspond to the results observed in other studies (Ferrie et al. 2013; Tüchsen et al. 2006; Niedhammer et al. 2014) including the meta-analysis (age- and sex-adjusted risk estimate of 1.32 for IHD) (Virtanen et al. 2013). Although more than 60 % of the study population took part in at least two DWECS waves, any misclassification of exposure for employees with only one measurement will probably bias the risk estimate toward the null. The Danish flexicurity model may be another reason for the low risk estimate. The Danish system with flexible rules for hiring and firing, social security, and active labor market policy (Madsen 2006) has resulted in high perceptions of job security (European Commission 2010).

Similar to the other studies (Virtanen et al. 2013; Levenstein et al. 2001), the risk was attenuated after multivariable adjustment for SES, shift work, and CVD risk factors (smoking status and BMI). Other psychosocial work factors (such as job strain) were differently assessed in the four survey waves and thus not included. Additional information on behavioral factors (leisure time physical activity), and clinical measurements were lacking in the present study. However, further adjustment for behavioral factors and physiological measures in the Whitehall II Study had little effect on the risk estimate (Ferrie et al. 2013). In addition, risk estimates after adjustments are difficult to interpret as work stress can lead to changes in health behavior and can thus mediate the association (Theorell 2014). Information on individual disposition (e.g., negative affectivity and coping styles) was not considered in previous cohorts (Virtanen et al. 2013) and in the present study.

Generally, the risk estimates after exploratory stratifications by age, and occupational status in the present study were comparable to other studies regarding inhomogeneous results, and problems with statistical power (Pejtersen et al. 2014), respectively. Depending on the outcome investigated, the presented gender-stratified analyses were inconsistent similar to previous cohort studies on job insecurity and IHD (Lee et al. 2004; Slopen et al. 2012; Netterstrøm et al. 2010; Virtanen et al. 2013) and treated hypertension (Levenstein et al. 2001. In the European cohorts, the difference in risk between men and women was not significant (Virtanen et al. 2013).

Employees under 50 years of age with perceived job insecurity filled 1.4 times more antihypertensive prescriptions than subjects without job insecurity. The risk for participants aged 50 years or more was not significant. The indicatively higher risk of younger employees may be explained by information from other sources on contextual factors of Denmark in the time period analyzed. The young Danish population in the 1990s suffered from high unemployment and less welfare benefits, compared with employees aged 60 years or more with a high proportion of early retirement due to almost universal access to voluntary retirement benefits in this age group (Kvist 2003). In the European cohorts (Virtanen et al. 2013), there was no evidence of significant differences in the association between perceived job insecurity and IHD by age. However, only one single measurement of job insecurity was available for a follow-up between one and more than 20 years.

The presented subanalysis with stratification by occupational group as an indicator of SES did not reveal a meaningful difference in antihypertensive medications dispensed to blue-collar workers with job insecurity as compared to white-collar workers with job insecurity. One could have anticipated a stronger reaction to perceived job insecurity among less-favored occupational groups due to economic dependency on paid work. However, the situation in the 1990s in Denmark was ambiguous with generous benefits for low-income groups (Kvist 2003). In the validation study, both incidence of IHD hospitalization/mortality and incident dispensing of antihypertensive medications were associated with the socio-occupational status (Hannerz et al. 2014). Low SES increased the incidence of IHD (Virtanen et al. 2013) and of treated hypertension with gender differences regarding different SES indicators (Levenstein et al. 2001). No stratification by SES was presented in the meta-analysis (Virtanen et al. 2013). Adjustment for SES attenuated the relation between job insecurity and IHD (Virtanen et al. 2013) as well as hypertension (Levenstein et al. 2001) indicating confounding or interrelated causal pathways. SES-related CVD risk factor profiles among employees with perceived job insecurity may be causal but may also be a consequence of fear of unemployment and thus mediate the association (Virtanen et al. 2013).

### Construct perceived job insecurity

Perceived job insecurity has been ascertained in different ways (Sverke et al. 2002) with either denial or probability of job security (Lee et al. 2004; Slopen et al. 2012; Ferrie et al. 2013) or affirmation of job insecurity with different operationalization of the COPSOQ job insecurity scale (Netterstrøm et al. 2010; Tüchsen et al. 2006; Rugulies et al. 2008, 2010) as in the present study. Subanalyses within the meta-analysis (Virtanen et al. 2013) implied an association only for the nine cohorts that assessed job insecurity/involuntary job loss and not for the six cohorts assessing the degree of job security.

Misclassification of the exposure due to the consideration of fear of job loss and/or anticipated poor reemployment opportunities in the present study would attenuate the risk estimates. The exploratory subanalysis of job insecurity strengthens the notion of conservative estimates in the main analysis. The subanalysis indicated a dose effect with the strongest association for subjects with perceived risk of involuntary job loss together with perceived lack of reemployment opportunities. The contrasting feature of the single-item global measurement of feared job loss might be increased by adding at least a second item on anticipated labor market chances if unemployed (Rugulies et al. 2008). Regarding physical health, a single item of perceived likelihood of involuntary job loss was as good as multidimensional instruments; the latter displayed stronger associations with psychosocial outcomes such as job satisfaction (Sverke et al. 2002). In order to comprehensively study the different adverse effects of job insecurity, multiple-indicator scales or at least all items from the COPSOQ job insecurity scale are necessary.

The risks of combined IHD morbidity or mortality as well as antihypertensive medication associated with perceived job insecurity were low in the present study and in the meta-analysis (Virtanen et al. 2013), as compared to the association of CVD with objective indicators of economic changes (Iversen et al. 1989; Vahtera et al. 2004). Organisational downsizing in Finland was associated with a doubled risk of death from CVD in employees who kept their jobs (Vahtera et al. 2004). Thus, future studies should additionally consider objective indicators of job insecurity.

### Outcome measurements

Even for a large cohort study with a long follow-up period, the present study was underpowered for the comparatively rare outcome of IHD (Pejtersen et al. 2014). In order to increase the statistical power, the dispensing of antihypertensive medications was analyzed as a second outcome. Hypertension is a relevant risk factor for IHD, and antihypertensive medication can prevent IHD (Perk et al. 2012). In the presented study, misclassification is likely because a wide scope of ATC codes (C02, C03, and C07–C09) was used in order to increase the sensitivity. These antihypertensive medications are also prescribed as treatment for other cardiovascular diseases (e.g., congestive heart failure, IHD, and arrhythmia) as well as other symptoms (e.g., migraine or palpation). However, hypertension or other cardiovascular diagnoses are the most frequent indications (Van Wijk 2006). In a validation study, Hannerz et al. (2014) demonstrated that dispensing of IHD-related medication is probably a useful indicator for IHD in the working population of Denmark. As there is no indication of differential misclassification, this proxy measure may lead to an underestimated risk estimate as compared to hospitalization or mortality. Further, observer and information bias are minimized for antihypertensive medications due to the Danish barcode labeling system for medications and the reimbursement procedures. In spite of guidelines recommendations, drugs are frequently used as the only measure to lower blood pressure (Perk et al. 2012). In a recent publication, dispensing of antihypertensive medication was also used to study a psychosocial work factor (Daugaard et al. 2014). However, not all subjects with hypertension are treated (Pereira et al. 2009; Gee et al. 2012), and adherence to antihypertensive treatment is related to demographic characteristics (Gee et al. 2012; Jensen and Schroll 2008). Higher SES in employees is related to less undetected hypertension, to higher adherence to therapy regimens (Gee et al. 2012), and to lower IHD morbidity and mortality characteristics (Tüchsen and Endahl 1999). Thus, the risk of blue-collar workers might be underestimated. Untreated subjects with hypertension have a higher probability of IHD. As a consequence, subjects in the present study were followed for both incidence of IHD and incident dispensing of antihypertensive medications with similar results. Further, perceived job insecurity increased the risk of antihypertensive prescriptions dispensed even after controlling for SES. Future studies should consider earlier outcomes such as medication or objective indicators of physiological stress reactions (Näswall et al. 2012) that predict IHD in order to minimize increasing statistical power. In addition, information on dosage and number of prescriptions could be utilized in future studies.

### Research needs

The present study and the meta-analysis (Virtanen et al. 2013) leave open questions regarding the best study design, mainly affected subgroups, and effective interventions.

Associated potential health risks should be investigated with cohort designs that consider multidimensional or objective measures with time-dependent changes in job insecurity in order to minimize misclassification of exposure. Less favorable subgroups suffer more from job insecurity, but statistical power is problematic (Pejtersen et al. 2014) given problems of misclassification of the exposure and the rare outcome of IHD/CVD. More frequently occurring indicators of the outcome such as incident chronic use of antihypertensive medication can increase the statistical power and allow for stratifications by socio-demographic indicators (e.g., age, gender, and occupational status) and contextual factors (e.g., unemployment rates, welfare benefits, and professional training options).

The available data for Denmark show that even within one country unemployment rates and the social support system changed over the 20-year follow-up. This necessitates further (multilevel) analyses with ascertainment of important aspects of job insecurity that incorporate both socio-demographic and contextual features (e.g., social support/benefits, and unemployment rates categorized by age and occupational status).

Given global changes with increasing workplace flexibility and growing temporary employment, stable occupational trajectories might be less expected among future generations. This warrants the evaluation of preventive measures such as increased opportunity of employees to participate in decision making regarding organisational changes (Vahtera and Virtanen 2013). Based on the present study, approaches involving continuous education in order to increase reemployment opportunities seem reasonable. Given that objective job insecurity, e.g., during an economic crisis, has a strong influence on the subjective fear of job loss (Chung and van Oorschot 2010), an analyses of the Danish version of flexicurity (Madsen 2006) and various case studies (e.g., Roche et al. 2011) demonstrate that the outcomes of an economic crisis or a restructuring process can be influenced in a positive way. A recent review demonstrates that effective and feasible intervention strategies to reduce occupational health disparities at the person (micro), workplace (meso) and/or policy (macro) level are available (Landsbergis et al. 2014). Further research is particularly needed in relation to the effectiveness of strategies on the macro-level (e.g., by employment protection legislation, active and passive labor market policy, and social dialog) and on the meso-level by organizational justice (transparent and fair decision process with employee participation) (e.g., Kieselbach 2009) on the health and well-being of disadvantaged worker groups.
